# The cuproptosis-related signature predicts prognosis and indicates immune microenvironment in breast cancer

**DOI:** 10.3389/fgene.2022.977322

**Published:** 2022-09-26

**Authors:** Jia Li, Fei Wu, Chaofan Li, Shiyu Sun, Cong Feng, Huizi Wu, Xi Chen, Weiwei Wang, Yu Zhang, Mengji Liu, Xuan Liu, Yifan Cai, Yiwei Jia, Hao Qiao, Yinbin Zhang, Shuqun Zhang

**Affiliations:** ^1^ Department of Oncology, The Second Affiliated Hospital of Xi’an Jiaotong University, Xi’an, China; ^2^ Department of Orthopedics, The Second Affiliated Hospital of Xi’an Jiaotong University, Xi’an, China

**Keywords:** cuproptosis, breast cancer, prognostic signature, tumor immune microenvironment, bioinformatics

## Abstract

Breast cancer (BC) is the most diagnosed cancer in women. Cuproptosis is new regulated cell death, distinct from known death mechanisms and dependent on copper and mitochondrial respiration. However, the comprehensive relationship between cuproptosis and BC is still blank until now. In the present study, we acquired 13 cuproptosis-related regulators (CRRs) from the previous research and downloaded the RNA sequencing data of TCGA-BRCA from the UCSC XENA database. The 13 CRRs were all differently expressed between BC and normal samples. Using consensus clustering based on the five prognostic CRRs, BC patients were classified into two cuproptosis-clusters (C1 and C2). C2 had a significant survival advantage and higher immune infiltration levels than C1. According to the Cox and LASSO regression analyses, a novel cuproptosis-related prognostic signature was developed to predict the prognosis of BC effectively. The high- and low-risk groups were divided based on the risk scores. Kaplan-Meier survival analysis indicated that the high-risk group had shorter overall survival (OS) than the low-risk group in the training, test and entire cohorts. GSEA indicated that the immune-related pathways were significantly enriched in the low-risk group. According to the CIBERSORT and ESTIMATE analyses, patients in the high-risk group had higher infiltrating levels of antitumor lymphocyte cell subpopulations and higher immune score than the low-risk group. The typical immune checkpoints were all elevated in the high-risk group. Furthermore, the high-risk group showed a better immunotherapy response than the low-risk group based on the Tumor Immune Dysfunction and Exclusion (TIDE) and Immunophenoscore (IPS). In conclusion, we identified two cuproptosis-clusters with different prognoses using consensus clustering in BC. We also developed a cuproptosis-related prognostic signature and nomogram, which could indicate the outcome, the tumor immune microenvironment, as well as the response to immunotherapy.

## Introduction

Breast cancer (BC) accounts for nearly one-third of all cancer cases in women, and its incidence rate increases by 0.5% each year (Siegel et al., 2022). According to the latest estimates, the new female BC cases will be 287,750, and deaths will be 43,250 in 2022 in the United States (Siegel et al., 2022). Histologically, BC includes three subtypes, including HER2-positive, endocrine-dependent, and triple-negative breast cancers (Maughan et al., 2010). Many therapeutic options have been developed, including surgery, chemotherapy, endocrine therapy, and anti-HER2 targeting. However, with standard diagnosis and treatment, it is estimated that 20–30% of patients with BC develop distant metastases, accounting for approximately 90% of the death of BC patients (Britt et al., 2020; Jabbarzadeh Kaboli et al., 2020). Furthermore, the considerable heterogeneity of tumors limits the broad applicability of typing and standard treatment to a certain extent (Waks and Winer, 2019). Thus, exploring the characteristics and potential therapeutic targets of BC patients is of great significance.

Cuproptosis is new regulated cell death (RCD), distinct from known death mechanisms and dependent on copper and mitochondrial respiration (Tsvetkov et al., 2022). Copper could bind to lipoylated components of the tricarboxylic acid (TCA) cycle, leading to lipoylated protein aggregation and subsequent iron-sulfur cluster protein loss, resulting in proteotoxic stress and ultimately cell death. The typical copper ionophores disulfiram (DSF) and elesclomol could induce copper delivery to intracellular compartments by ionophore to induce cuproptosis and are being used as anticancer and chemosensitizing effects in cancer therapeutics (Gehrmann, 2006; Liu et al., 2013; Viola-Rhenals et al., 2018; Yang et al., 2021). Previous studies on copper have focused on the disturbances of copper homeostasis, which is related to a series of diseases, such as Menkes disease (Horn and Wittung-Stafshede, 2021) and Wilson disease (Członkowska et al., 2018). Moreover, elevated copper concentrations have been reported in many cancers, such as breast (Adeoti et al., 2015), lung (Oyama et al., 1994), prostate (Saleh et al., 2020), thyroid (Baltaci et al., 2017), gastrointestinal (Stepien et al., 2017) and gall bladder (Basu et al., 2013) cancers. The role of copper in cancers might partly be promoting blood vessel formation, tumorigenesis, and metastasis (Shanbhag et al., 2019). Many drugs have been developed to regulate copper metabolism in the body for those copper imbalance diseases. Copper chelators could act as an antiangiogenic treatment in many cancers (Brewer et al., 2000; Sen et al., 2002; Chan et al., 2017), regulate autophagy (Krishnan et al., 2018; Bryant et al., 2019), modify the tumor microenvironment (Chan et al., 2017), and enhance the antitumor immunity (Voli et al., 2020).

Cuproptosis could be regulated by specific genes: cuproptosis-related regulators (CRRs), including DLD, PDHB, ATP7B, ATP7A, DLAT, DLST, SLC31A1, DBT, FDX1, LIPIT1, LIAS, GCSH, and PDHA1 (Tsvetkov et al., 2022). Further research into these CRRs could help us understand cuproptosis in disease. Mounting evidence has revealed that signatures based on cell death patterns showed substantial predictive values in prognostic, tumor immune microenvironment (TIME), and immunotherapy response prediction for BC patients, such as ferroptosis (Zhu et al., 2021a), pyroptosis (Xu et al., 2022), and necroptosis (Hu et al., 2022), but studies on the role of cuproptosis in BC lack to some extent. An in-depth study about the association between the genetic changes and expression dysregulations of the CRRs and tumor will be beneficial for the identification of the role of cuproptosis in BC and new therapeutic targets.

Our research comprehensively explored the expression variations, genetic changes, and functions of CRRs in BC. We used consensus clustering analysis to identify two cuproptosis-clusters with different overall survival (OS) and TIME characteristics. We also developed a cuproptosis-related prognostic signature and nomogram, which could indicate the outcome, the tumor immune microenvironment, as well as the response to immunotherapy.

## Material and methods

### Data acquisition

**FIGURE 1:**
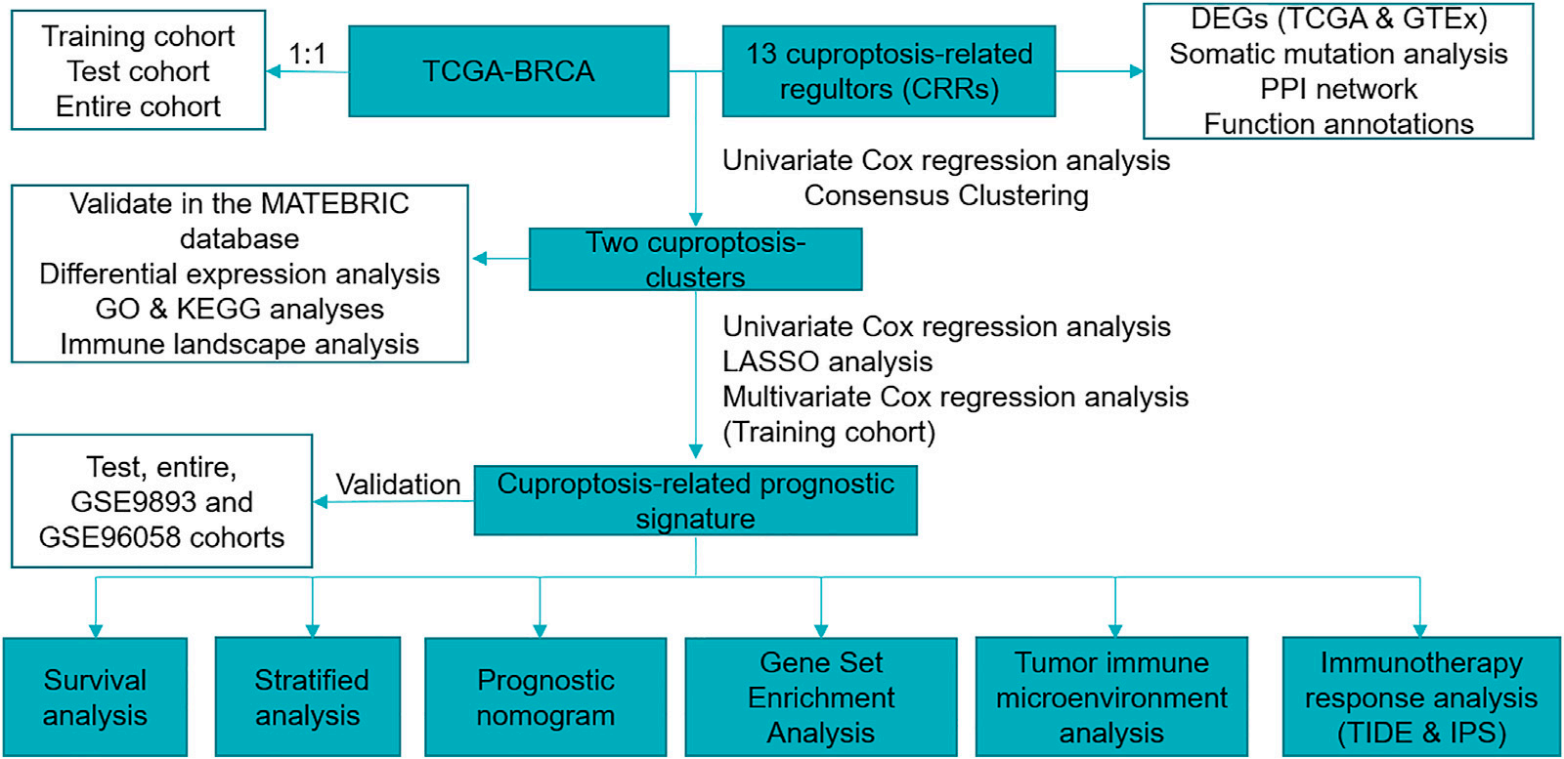
Workflow of the current study.

The RNA sequencing data of BC and normal samples in The Cancer Genome Atlas (TCGA) (113 normal breast samples and 1,109 BC samples and Genotype-Tissue Expression (GTEx) database were downloaded from the UCSC XENA (https://xenabrowser.net/datapages/). The Molecular Taxonomy of Breast Cancer International Consortium (METABRIC) database (Pereira et al., 2016) was downloaded from cBioportal (http://www.cbioportal.org/) (Cerami et al., 2012), and 1,758 of 1,904 BC patients with OS > 30 days were used for analysis. The microarray dataset GSE9893 (N = 155) and GSE96058 (N = 3,069) were downloaded from GEO (http://www.ncbi.nlm.nih.gov/geo/). We used log2 (TPM) data to evaluate the expression of CRRs between BC and normal samples. We excluded male patients and the patients with OS < 30 days and finally remained 916 female patients for further study. The “caret” R package randomly divided our sample into two cohorts at a 1:1 ratio (training and test). The “tableone” R package analyzed the clinical features of the training, test, and entire cohorts (Supplementary Table S1). In the subsequent clinicopathological correlation analysis, we excluded patients with incomplete information. We acquired 13 cuproptosis-related regulators (CRRs) from the previous investigation (Supplementary Table S2) (Tsvetkov et al., 2022). Figure 1 showed the workflow of the current study.

### Consensus clustering for cuproptosis-clusters

Univariate Cox regression analysis was used to evaluate the CRRs with prognostic values. We used the R package “ConsensusCluster Plus” to perform consensus clustering analysis and identify cuproptosis-clusters in BC patients. We set cluster count (k) between 2 and 9 and selected the optimal k value based on the inflection point of the sum of squared error (SSE). The stability of cuproptosis-clusters was verified by the t-distributed stochastic neighbor embedding (tSNE) algorithm. Furthermore, the Kaplan-Meier survival analysis evaluated the OS of the different cuproptosis-clusters. We further validated the cuproptosis-clusters with the same consensus clustering analysis in the METABRIC data.

### Identification of differentially expressed genes and functions of the cuproptosis-clusters

We performed “edgeR” R package to analyze the DEGs between the two cuproptosis-clusters (*p* < 0.05, |logFC| = 1). We used Gene Ontology (GO) and Kyoto Encyclopedia of Genes and Genomes (KEGG) to annotate the functions of DEGs with the R package “ClusterProfiler” (Yu et al., 2012).

### Construction and validation of the cuproptosis-related prognosis signature

We retained 336 DEGs expressed in more than half of the patients for further study for the accuracy of the results. These DEGs were submitted to univariate Cox regression analysis to identify the prognostic DEGs in TCGA-training and GSE9893 cohorts. The least absolute shrinkage and selection operator (LASSO) was used to avoid overfitting in the TCGA-training cohort (Friedman et al., 2010). Then, the cuproptosis-related prognostic signature (CRPS) was built with the multivariate Cox regression analysis and stepwise Akaike information criterion (stepAIC) value. Subsequently, each sample could get the risk score according to the formula: Risk score = Σ(Exp∗ Coef). The Coef and Exp were the coefficients and the expression level of each gene, respectively. Based on the median risk score of the training cohort, we divided patients into high- and low-risk groups. For the training, test, entire, GSE9893 and GSE 96058 cohorts, Kaplan-Meier survival analysis and time-dependent receiver operating characteristic (ROC) curves were used to assess the predictive accuracy of the CRPS. We further performed the stratified analyses to assess the prognostic value of the CRPS in different subgroups stratified by age, pathologic stage, T stage, N stage, M stage, ER, PR, and HER2 statuses.

### Clinical analysis and construction of the nomogram

We compared the risk score in different clinical characters, including stage, PAM50 subtypes, ER, PR and HER2 status. Multivariate COX regression analysis was performed to assess the independent prognostic factors. The nomogram was constructed with the independent prognostic factors. The calibration curves assessed the accuracy of the nomogram.

### The different tumor immune microenvironment patterns between the two risk groups

The variations of pathway activity of the two risk groups were revealed with Gene Set Enrichment Analysis (GSEA) (*p* < 0.05 and FDR< 0.25) (Subramanian et al., 2007). The annotated gene set “c2. cp.kegg.v7.5.1. symbols.gmt” was acquired from the MSigDB (https://www.gsea-msigdb.org/gsea/msigdb/). The CIBERSORT algorithm calculated the proportion of tumor-infiltrating immune cells (Newman et al., 2015). Immune, stromal, and tumor purity scores were evaluated with the ESTIMATE algorithm (Yoshihara et al., 2013). In assessing immune response, well-known predictors of immune checkpoints (ICPs) have been used extensively. Therefore, we compared the tumor-infiltrating immune cells, immune and stromal scores, and 27 ICPs in the two risk groups.

### Prediction of the response to immune checkpoint inhibitors treatment

The immunophenoscore (IPS) represents a comprehensive measure of immunogenicity and could predict the patient’s response to ICIs treatment (Charoentong et al., 2017). We acquired the IPS score of 916 BC patients from The Cancer Immunome Atlas (https://tcia.at/) and compared the potential immunotherapy in the two risk groups. TIDE provided an easy way to predict the response to ICIs based on evaluating the tumor microenvironment (http://tide.dfci.harvard.edu/) (Jiang et al., 2018; Fu et al., 2020). After submitting the transcriptome profiles of 916 TCGA-BRCA patients to the website, we acquired the information on whether the patients could respond to the ICIs.

### Statistical analysis

We applied R software (version 4.0.5, https://www.r‐project.org/) for all statistical analyses. Comparative analysis between groups was performed using Student’s t-test, Wilcoxon test, or one-way variance analysis. *p*-value < 0.05 was set as statistically significant, and the significance levels were set as *p < 0.05, **p < 0.01, ***p < 0.001, ****p < 0.0001, ns: nonsignificant.

## Results

### Overview of expression variations, genetic changes and function analyses of CRRs in BC

**FIGURE 2:**
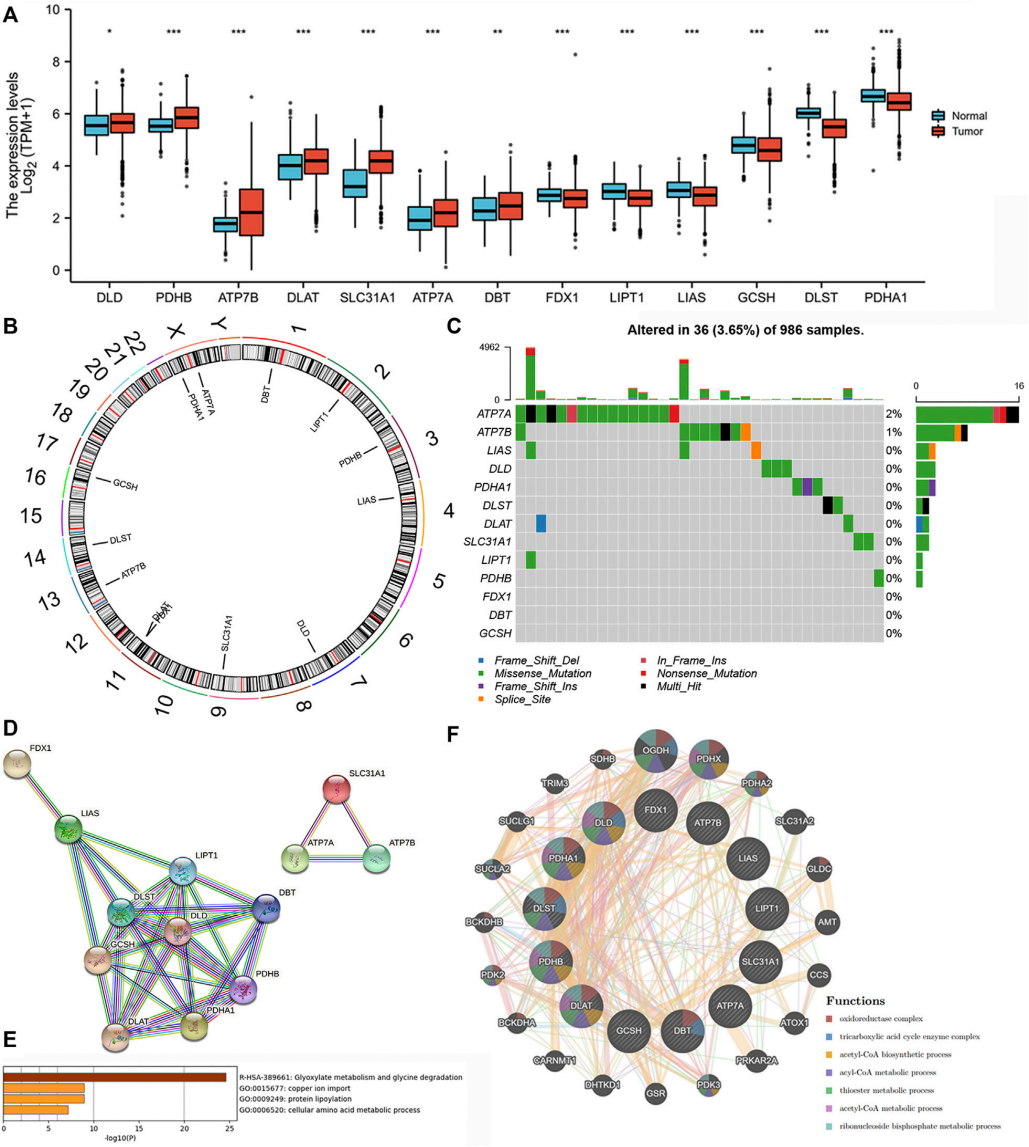
Expression variations, Genetic changes, and functional analyses of CRRs in BC. **(A)** The expression of CRRs between BC and normal samples. **(B)** The location of CRRs on chromosomes. **(C)** The schematic overview of mutation frequency and type in CRRs. **(D)** PPI network of the CRRs-encoded proteins. **(E)** The GO analysis of CRRs in the METASCAPE database. **(F)** The network and function of CRRs in the GENEMANIA database.

We found that all of the 13 CRRs were DEGs between BC and normal samples, among which seven regulators (DLD, PDHB, ATP7B, DLAT, SLC31A1, ATP7A, DBT) were upregulated and six regulators (FDX1, LIPT1, LIAS, GCSH, DLST, PDHA1) were downregulated in BC samples (Figure 2A). Figure 2B shows the location of 13 CRRs on the chromosome. The genetic analysis revealed that 36 of the 986 samples (about 3.5%) carried mutations in the regulator of cuproptosis, ATP7A exhibited the highest frequency of mutations, and the majority of mutations were missense mutations. There were no FDX1, DBT, or GCSH mutations in the BC samples (Figure 2C). To better understand the mode of interaction between these CRRs, the protein-protein interaction (PPI) network retained all 13 CRRs with complex regulatory relationships at a high confidence score (0.7) (Figure 2D). We investigated the biologic function and behavior of the 13 CRRs using Metascape and GENEMINIA enrichment analysis. The results of the Metascape database indicated that the 13 CRRs were significantly enriched in the following terms: Glyoxylate metabolism and glycine degradation, copper ion import, protein lipoylation, and cellular amino acid metabolic process (Figure 2E). Furthermore, they were associated with oxidoreductase complex, tricarboxylic acid cycle enzyme complex, acetyl-CoA biosynthetic process, ancl-CoA metabolic process, thioester metabolic process, acetyl-CoA metabolic process, ribonucleoside bisphosphate metabolic process based on the GENEMIAIA database (Figure 2F). According to our analysis, the CRRs were related to cuproptosis, and the expression levels of CRRs were correlated with BC, suggesting that cuproptosis might reflect different traits in patients.

### Identification of the cuproptosis-clusters with CRRs in BC

**FIGURE 3:**
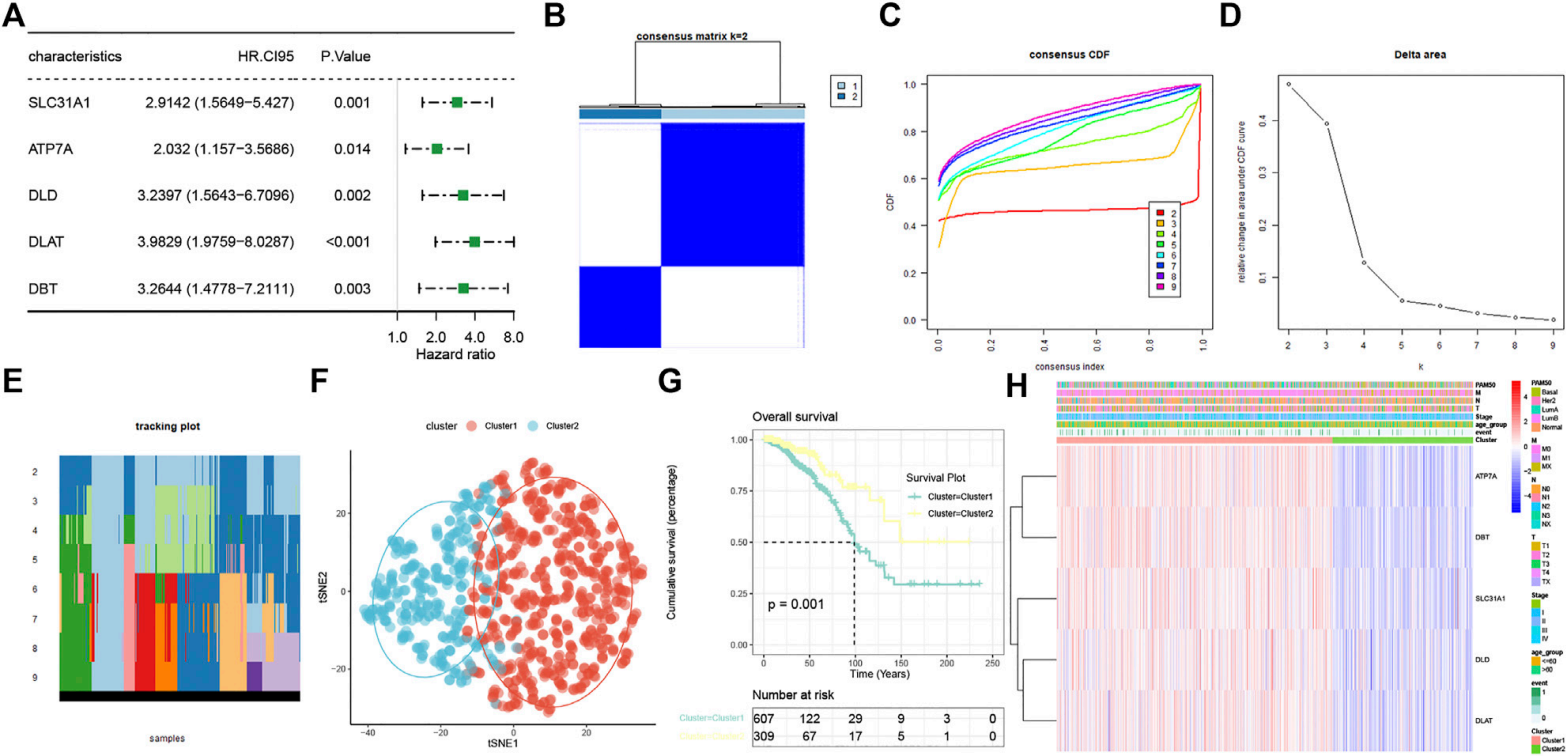
Characterization of two cuproptosis-clusters based on CRRs in BC. **(A)** Univariate Cox regression analysis of CRRs. **(B)** Consensus matrix when k = 2. **(C)** Consensus CDF, **(D)** Delta area, **(E)** tracking plot, and tSNE plots **(F)** for validation of the clustering results. **(G)** Kaplan-Meier OS curves for BC patients between C1 and C2. **(H)** Unsupervised clustering of CRRs in the TCGA-BRCA cohort. The cuproptosis-clusters, stage, age, TNM stage, PAM50 subtypes, and survival status were used as patient annotations.

The prognostic values of the 13 CRRs were analyzed with univariate Cox regression analysis, and five (SLC31A1, ATP7A, DLD, DLAT, and DBT) were significantly associated with BC patient prognosis (Figure 3A). The consensus clustering analysis based on these five CRRs explored the relationship between cuproptosis and BC subtypes. Among the clustering variables, k = 2 showed excellent clustering stability with the highest intragroup correlations and the lowest intergroup correlations. Therefore, we acquired two cuproptosis-clusters (C1 and C2) (Figures 3B–D). Figure 3E revealed that C1 was significantly different from C2 (tSNE). Moreover, C2 had a more favorable OS than those C1 (Figure 3F). As shown in Figure 3G, the two cuproptosis-clusters exhibited different gene expression profiles and clinical features. Surprisingly, all five CRRs elevated in C1. To validate the cuproptosis-clusters in BC, we further performed the same consensus clustering analysis in the METABRIC database, and the results indicated that 1758 BC patients were clustered into two clusters (Supplementary Figures S1A–D). Cluster 1 significantly differed from cluster 2 (tSNE) and had a worse OS than cluster 2 (Supplementary Figures S1E,F). These results indicated that we successfully identified two cuproptosis-clusters of breast cancer.

### The cuproptosis-clusters characterized with different immune profiles

**FIGURE 4:**
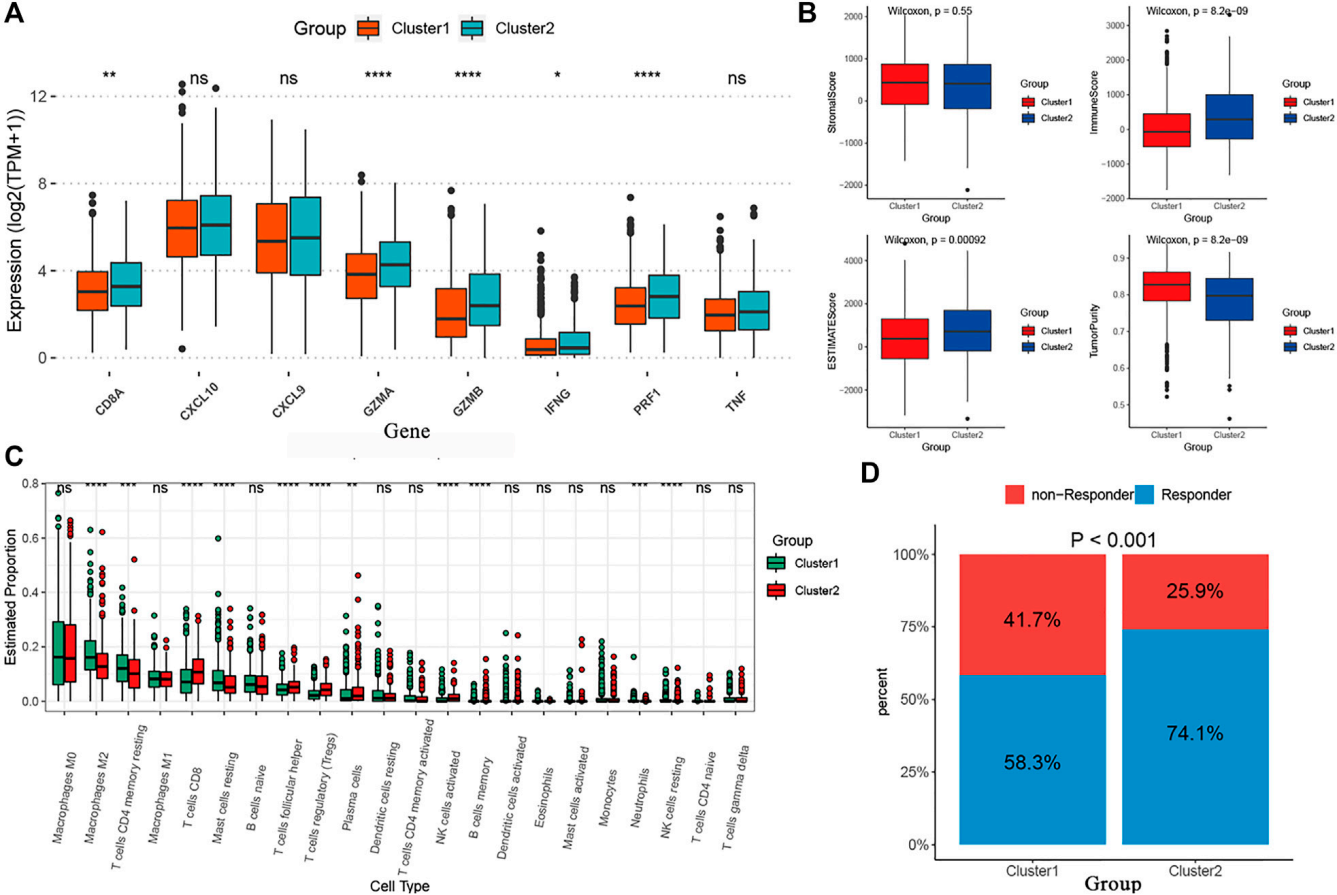
The different immune profiles between the two cuproptosis-clusters. **(A)** Comparisons of the immune-activation related gene expression between the two cuproptosis-clusters. **(B)** Comparisons of tumor purity, stromal, immune and estimate scores between the two clusters. **(C)** The box plots of the tumor-infiltrating cells in the two clusters. **(D)** Comparisons of the proportions of non-responders and responders to immunotherapy between the two risk clusters.

We performed the “edgeR” R package and identified 4891 DEGs between the two curproptosis-clusters (Supplementary Figures S2A). The GO and KEGG analyses of the DEGs showed that immune activation pathways were enriched in C2, such as regulation of humoral immune response, cytokine–cytokine receptor interaction, and IL-17 signaling pathway (Supplementary Figures S1B,C). We further performed a series of immune-related analyses. We found that some transcripts of immune activation, such as IFNG, GZMB, CD8A, PRF1, and GZMA expressed higher in C2 than in C1 (Figure 4A). According to the CIBERSORT algorithm, many antitumor lymphocyte cells subpopulations were significantly elevated in C2, such as activated CD4^+^/CD8^+^ T cells, NK cells, and plasma. In contrast, M2 macrophages were elevated in the C1 (Figure 4B). The ESTIMATE analysis further revealed that C2 had higher immune and ESTIMATE scores while C1 had higher tumor purity (Figure 4C). The results of TIDE indicated that C2 had a higher proportion of responders to immunotherapy (Figure 4D). These results indicated that the two cuproptosis-clusters characterized with different immune landscapes.

### Construction and validation of the cuproptosis-related prognostic signature

**FIGURE 5:**
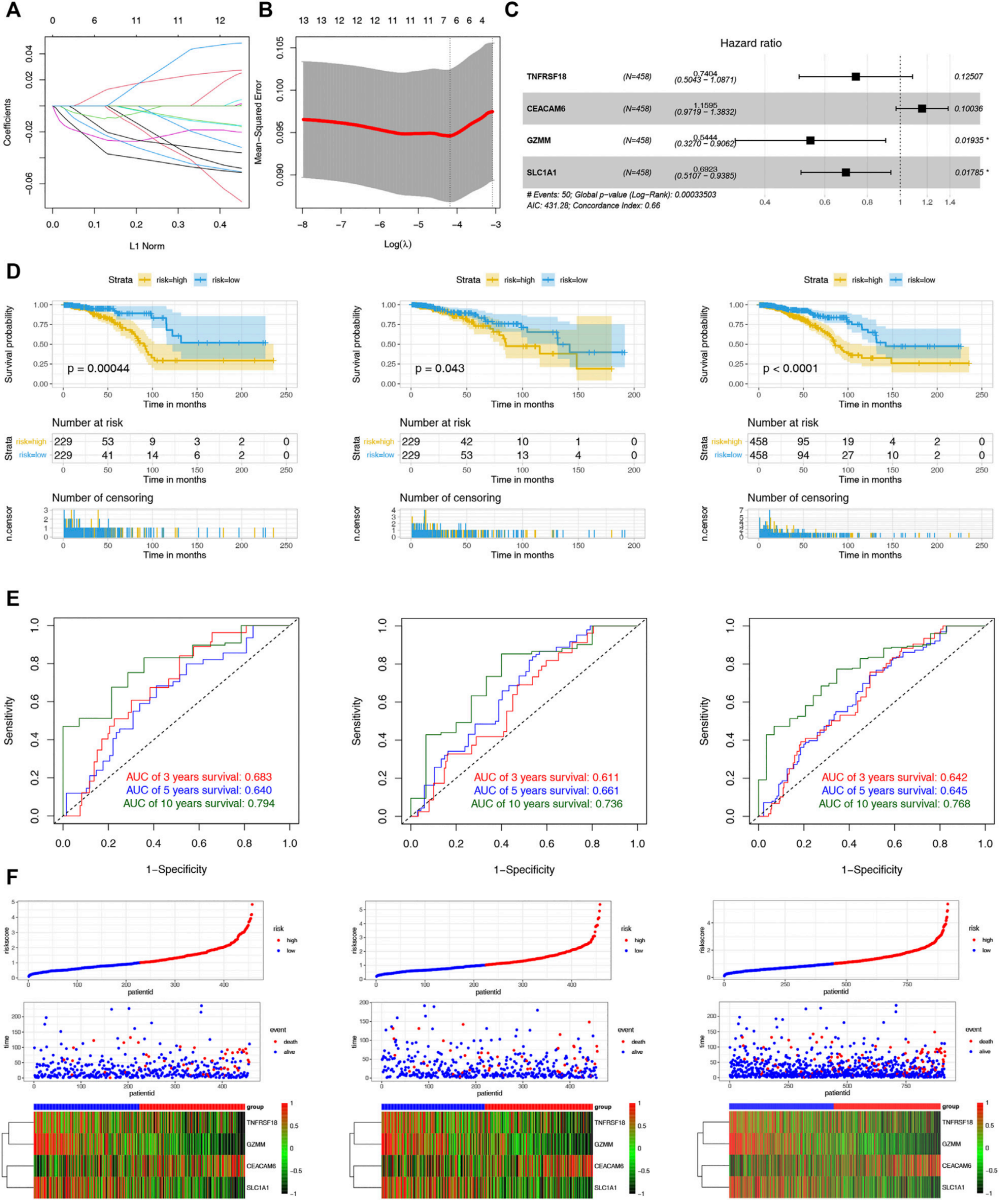
Construction and Validation of the CRPS. **(A)** LASSO regression analysis of 108 prognostic DEGs. **(B)** Cross validation method to select optimal genes. **(C)** The forest plot of multivariate Cox regression analysis. **(D)** Kaplan–Meier analyses of the OS between the two risk groups in the training, test, and entire cohort. **(D)**The 3-, 5- and 10-year ROC curves of the CRPS in the training, test, and entire cohort. **(F)** Ranked dot, scatter plots and heat map of the model gene expressions in the training, test, and entire cohort.

To assist clinicians in predicting the prognosis of BC patients, we constructed the CRPS. Using univariate Cox regression analysis, we identified 16 cuproptosis-related DEGs associated with prognosis for BC in the TCGA-training and GSE9893 cohorts (Supplementary Table S3). The LASSO regression algorithm determined six OS-related genes based on the optimum λ value and the minimum partial likelihood of deviance (Figure 5A,B). The six genes were submitted to the multivariate Cox regression analysis. CRPS was formed by incorporating four genes based on AIC values, and its formula is listed below: Risk score = (-0.3005824 * TNFRSF18) + (-0.3676786* SLC1A1) + (0.1479489 * CEACAM6) + (-0.6081390 * GZMM) (Figure 5C). Patients with BC were categorized into low- and high-risk groups using the median risk scores from the training cohort. Based on the survival analyses, the high-risk group experienced earlier death and a poorer outcome than the low-risk group (Figure 5D). Across the training, test and entire cohorts, the AUCs for the 10-year ROC were 0.794, 0.736, and 0.768, respectively (Figure 5E). Figure 5F showed the distribution plot of the risk score and the different expression levels of the model genes in the two risk groups. We further performed the same analyses in the GSE9893 and GSE96058 databases. The low-risk group had a worse OS than the high-risk group (Supplementary Figures S1A,B). We further selected the appropriate time according to the follow-up characteristics of different cohorts to draw the ROC curves for verification. In GSE9893 database, the AUCs of the 4-, 5-, and 10-year ROC curves were 0.608, 0.695 and 0.593 (Supplementary Figures S3C). In GSE96058 database, the AUCs of the 3-, 5-, and 6-year ROC curves were 0.6065, 0.6215 and 0.6216 (Supplementary Figures S3D). The expression levels of the model genes in the two risk groups were highly similar to the TCGA cohorts (Supplementary Figures S3E,F). Considering the heterogeneity of BC, stratified analyses were further used to assess the prognostic value of CRPS in different clinical subgroups. The results revealed that patients in the low-risk group exhibited a prominent survival benefit compared with the high-risk group in most clinical subgroups, except for luminal B subtypes (Supplementary Figures S4). The above results indicated the prognostic signature was accurate, independent and widely applicable.

### Evaluation of the clinical significance of CRPS and development of the cuproptosis-based prognostic nomogram

**FIGURE 6:**
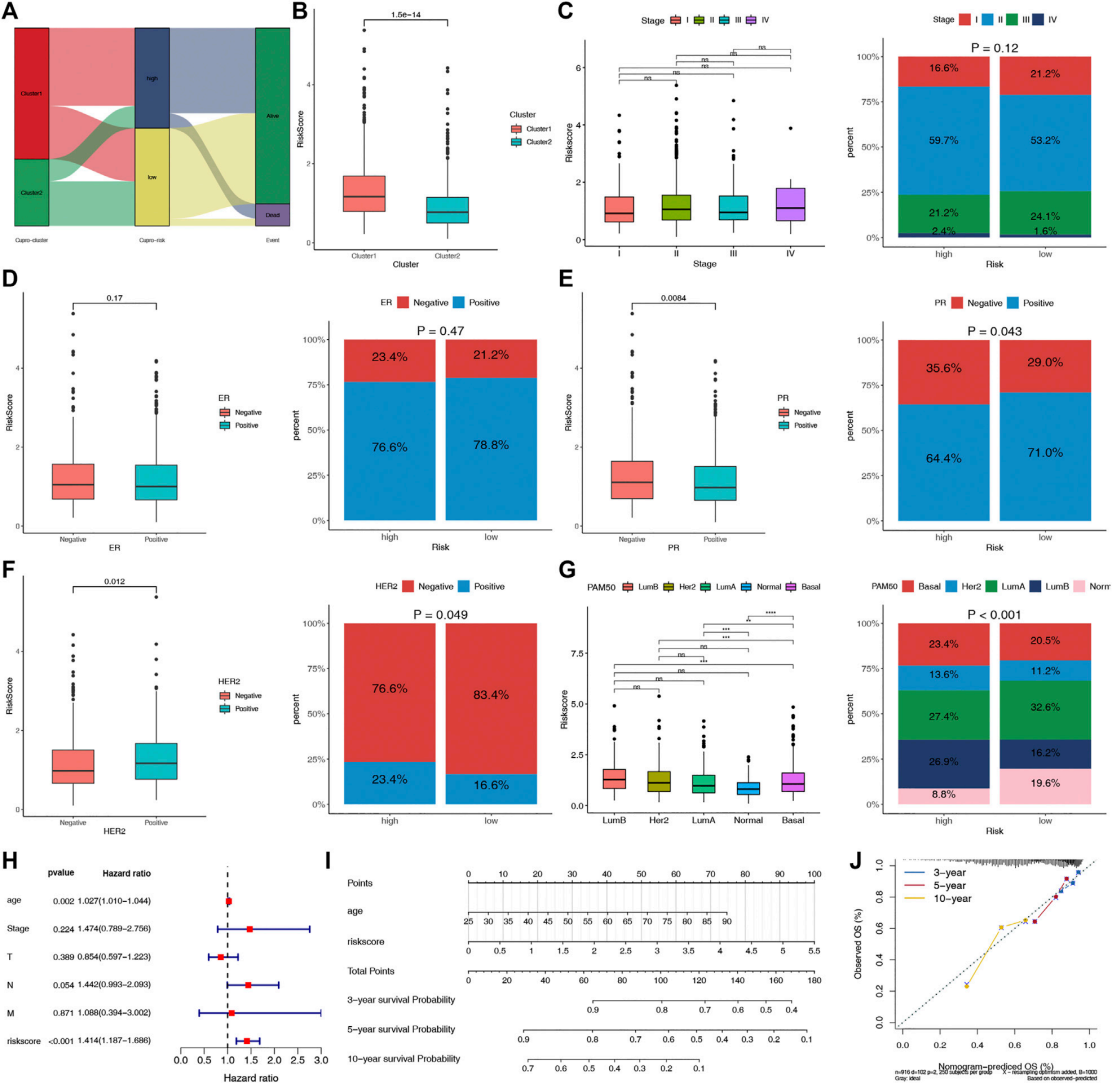
Evaluation of the clinical significance of CRPS and development of the cuproptosis-related prognostic nomogram. **(A)** Alluvial diagram of cuproptosis-clusters, risk groups and outcome. **(B)** Comparisons of the risk score in the two cuproptosis-clusters. **(C–G)** The different phenotype Relationships of the risk score and Stage, ER, PR, HER2, PAM50 phenotypes, respectively. **(H)** Multivariate Cox regression analysis of clinical characteristics and risk score. **(I)** The nomogram for predicting BC patients’ 3-, 5-, and 10-year OS probability. **(J)** The 3-, 5-, and 10-year calibration curves of the nomogram.

The potential clinical utility of the risk score of CRPS was further analyzed. The alluvial diagram showed the relationship between cuproptosis-cluster, risk groups and outcomes (Figure 6A). C1 was linked to the high-risk group and death, while C2 was related to the low-risk group and living. Moreover, C1 exhibited a higher risk score than C2 (Figure 6B). HER2-and PR+ were related to the low-risk score, and basal subtype was correlated with the high-risk score (Figures 6C–G). Furthermore, multivariate COX regression analysis revealed that age and the risk score were the independent prognostic factors for BC patients (Figure 6H). We further established the prognostic nomogram to quantitatively predict the 3-, 5- and 10-year survival probability of BC patients (Figure 6I). The calibration curve of the nomogram demonstrated that it could predict the survival probability relatively well (Figure 6J).

### Comparison with other prognostic signatures

The robustness of our CRPS was assessed by comparing it with 13 existing OS‐related prognostic signatures, such as ferroptosis, apoptosis, pyroptosis, necroptosis, immunity, and zinc finger proteins. In order to eliminate the effect of heterogeneity, we included only signatures generated using the TCGA database. AUC was used to assess the predictive power of signatures, and a larger AUC indicates better classification ability (Fawcett, 2006), and it could be used to compare the performance of the signatures (Zhang et al., 2021; Lv et al., 2022). We integrated all the important information of the thirteen signatures, including author, year, gene signature and the AUCs of the signatures (Table 1). Our signature had many advantages in predicting OS in BC patients, especially for the 10-year OS. In our study, the AUCs of the signatures at 3-, 5-, and 10-year were 0.683, 0.640, and 0.794, respectively, significantly higher than most hallmark predictive models. Table 1 showed that the 3-, 5-, and 10-year AUCs of the other four prognostic signatures, namely, the 9 ferroptosis-related gene signature (3-, 5-, and 10-year AUCs: 0.713, 0.713, and 0.684) (Lu et al., 2022a), 4 immune-related gene signature (3-, 5-, and 10-year AUCs: 0.692, 0.691, and 0.715) (Ding et al., 2021), 7 pyroptosis-related gene signature (3-, 5-, and 10-year AUCs: 0.785, 0.769, and 0.711) (Chu et al., 2022), and 6 autophagy-related gene signature (3-, 5-, and 10-year AUCs: 0.640, 0.600, and 0.610) (Zhong et al., 2020) were comparable to the predictive capabilities of our predictive model, and our signature stand out with a clear advantage in predicting the long-term survival of BC patients, with a 10-year AUC of 0.819. We also listed the other signatures that focus more on short-term survival, such as 1-year, 2-year, 3-year, and 5-year survival. We found our signature have similar short-term survival (3-year) prognostic value compared with them, such as the ferroptosis-related gene signature (Wang et al., 2021a; Wu et al., 2021a; Zhu et al., 2021b), pyroptosis-related gene signature (Chen et al., 2022), necroptosis-related gene signature (Yu et al., 2022), apoptosis-related gene signature (Zou et al., 2022), zinc finger protein-related gene signature (Ye et al., 2021), autophagy-related gene signature (Lin et al., 2020), and metabolic-related gene signatures (Lu et al., 2022b). In addition, our model only involves four genes, while other models (11/13) tend to have more, which is more convenient to use to a certain extent. These results indicated that our gene signature outperformed most other signatures in predicting BC prognosis.

**TABLE 1:** The area under the ROC curve (AUC) showed the sensitivity and specificity of the known gene signatures in predicting the prognosis of BC patients.

Author	Year	Gene signature	Gene number in signature	AUC for OS
Our study	2022	Cuproptosis	4	0.683 (3-year), 0.640 (5-year), 0.794 (10-year)
Lu, et al. (2022a)		Ferroptosis	9	0.713 (3-year), 0.713 (5-year), 0.684 (10-year)
Ding et al. (2021)		Immune	4	0.692 (3-year), 0.691 (5-year), 0.715 (10-year)
Ling et al. (2022)		Pyroptosis	7	0.785 (3-year), 0.769 (5-year), 0.711 (10-year)
Zhong et al. (2020)		Autophagy	6	0.640 (3-year), 0.600 (5-year), 0.610 (10-year)
Zhu et al. (2021)		Ferroptosis	11	0.700 (1-year), 0.749 (2-year), 0.720 (3-year)
Ding et al. (2021)		Ferroptosis	9	0.618 (1-year), 0.653 (2-year), 0.663 (3-year)
Wu et al. (2021a)		Ferroptosis	15	0.740 (1-year), 0.710 (3-year), 0.750 (5-year)
Chen et al. (2022)		Pyroptosis	16	0.756 (1-year), 0.752 (3-year), 0.723 (5-year)
Yu et al. (2022)		Necroptosis	6	0.701 (1-year), 0.716 (2-year), 0.708 (3-year)
Zou et al. (2022)		Apoptosis	6	0.708 (1-year), 0.628 (3-year), 0.687 (5-year)
Min et al. (2021)		Zinc finger protein	14	0.732 (1-year), 0.768 (2-year), 0.737 (3-year)
Lin et al. (2020)		Autophagy	12	0.739 (1-year), 0.717 (2-year), 0.742 (3-year)
Lu et al. (2022b)		Metabolic	2	0.764 (1-year), 0.689 (3-year), 0.612 (5-year)

### Different immune landscapes in the two risk groups

**FIGURE 7:**
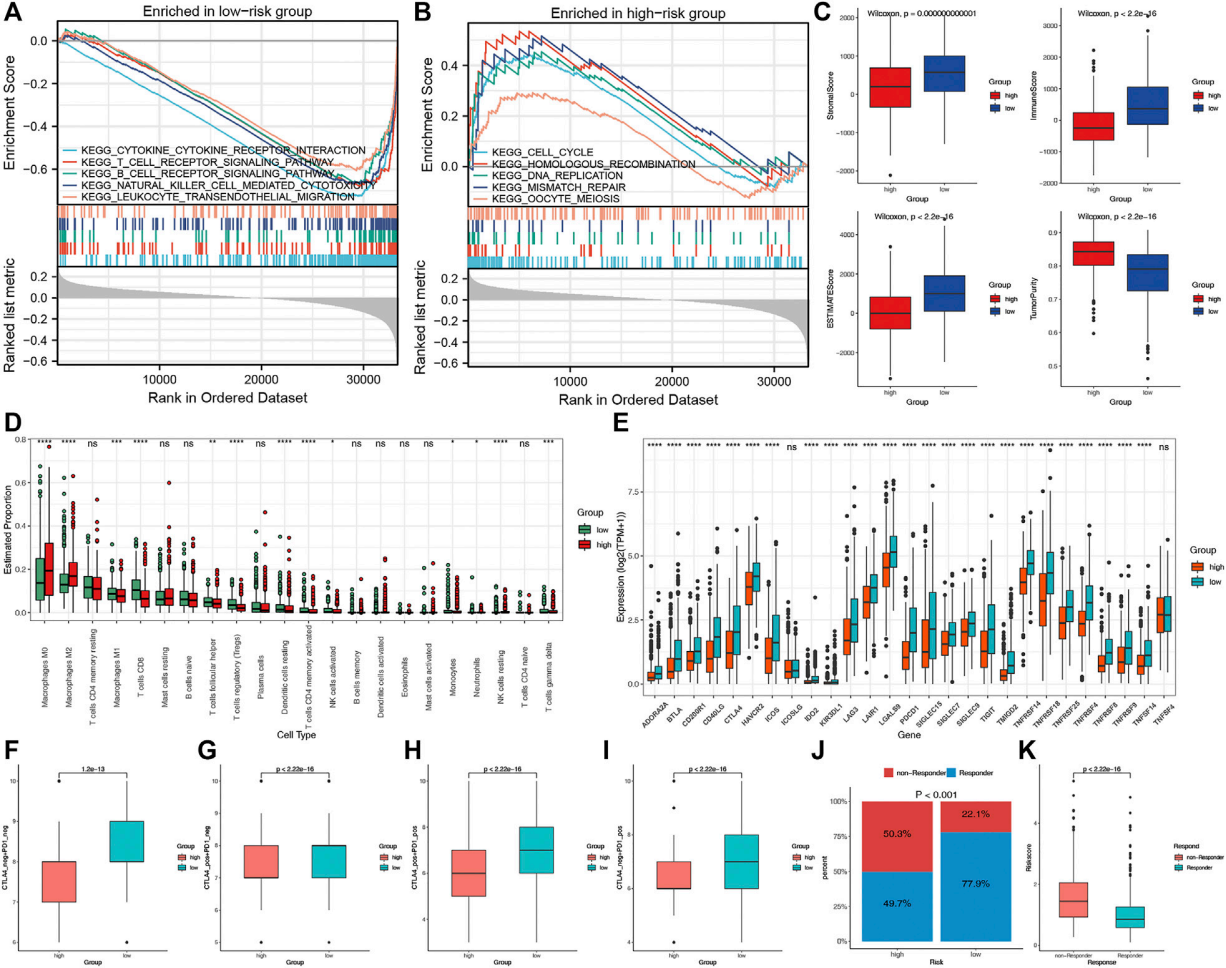
The TIME and immunotherapy response between the two risk groups. Significantly enriched pathways in low-risk **(A)** and high-risk **(B)** groups according to the GSEA. **(C)** Comparisons of tumor purity, stromal, immune and estimated scores between the two risk groups. **(D)** The box plots of the tumor-infiltrating cells in the two risk groups. **(E)** The expression levels of 27 ICPs in the two risk groups. **(F–I)** Relative distribution of immunotherapeutic efficacy in the two groups. **(J)** Comparisons of the proportions of non-responders and responders to immunotherapy between the two risk groups. **(K)** Boxplot showed a significantly higher risk score in the responders.

To explore the potential biological processes of the two risk groups, we performed GSEA. Interestingly, a series of immune-related pathways were enriched in the low-risk group, while cell cycle and tumor-related pathways were enriched in the high-risk group (Figures 7A,B). We subsequently used CIBERSORT and ESTIMATE analyses to analyze the TIME of the two risk groups. The ESTIMATE results revealed that the high-risk group had lower stromal, immune, and estimate scores but had higher tumor purity (Figure 7C). The CIBERSORT analysis further indicated that the macrophages M2, M0 and NK cells resting were the main components of the high-risk group; however, most of the antitumor lymphocyte cells, such as macrophages M1, CD8 T cells, dendritic cells resting, and activated NK cells were the main components of the low-risk group (Figure 7D). Furthermore, the expression levels of the 27 common ICPs were significantly elevated in the low-risk group, such as PD-1, PD-L1 and CTLA4 (Figure 7E). These results indirectly indicated the different immune landscapes in the two risk groups.

### Different immunotherapy responses in the two risk groups

We evaluated the immunotherapy response by the IPS and TIDE. Patients in the low-risk group presented significant therapeutic benefits from ICIs treatment according to the IPS (CTLA4-/PD-1-, CTLA4+/PD-1-, CTLA4-/PD-1+ and CTLA4+/PD-1+) (Figures 7F–I). Based on the TIDE analysis, the responders to immunotherapy in the low-risk group were more than in the high-risk group (77.9 versus 49.7%) (Figure 7J). Furthermore, the risk score was significantly elevated in non-responders to immunotherapy than in responders (Figure 7K). These results revealed that the essential role of CRPS in predicting the response to immunotherapies.

## Discussion

Recently, cuproptosis has been identified as a novel RCD (Tsvetkov et al., 2022). It depends on copper and mitochondrial respiration. Previous studies indicated that cuproptosis could be induced by copper ionophores drugs DSF and elesclomol and had anticancer and chemosensitizing effects (Gehrmann, 2006; Liu et al., 2013; Viola-Rhenals et al., 2018; Yang et al., 2021). The importance of other RCDs had been revealed in BC, but the role of cuproptosis in BC is still unknown (Zhu et al., 2021a; Hu et al., 2022; Xu et al., 2022). Our research was aimed at exploring the importance of cuproptosis in predicting the prognosis, TIME, and immunotherapy response for BC patients, which might lay the foundation for precise cuproptosis-related treatment of BC.

In our study, we firstly revealed the expression patterns, genetic alterations and biological functions of CRRs in BC. We found that all 13 CRRs were DEGs between BC and normal samples. The low mutation rates indicated the maintenance of genome stability. The functional analysis of these 13 CRRs revealed that they were enriched in the biological process of cuproptosis, including copper ion import, tricarboxylic acid cycle enzyme complex, and thioester metabolic process. However, only five CRRs (SLC31A1, ATP7A, DLD, DLAT, and DBT) had the prognostic value and were used for consensus clustering analysis. Among these five CRRs, SLC31A1 was associated with chemoresistance to platinum in osteosarcoma (Cheng et al., 2020), lung cancer (Wang et al., 2021b), and ovarian cancer (Wu et al., 2021b). The copper efflux transporter ATP7A was related to the chemoresistance in esophageal and ovarian cancers (Lukanović et al., 2020; Li et al., 2021). These results showed the significance of CRRs in regulating the occurrence, development and treatment of tumors.

Consensus Clustering is a common method of classifying cancer subtypes (Zhang et al., 2020; Wu et al., 2021c; Han et al., 2021). We performed consensus clustering with the five prognostic CRRs in TCGA-BRCA data and two distinct cuproptosis-clusters were identified. The OS of BC patients were strongly different between the two cuproptosis-clusters. The stability of the cuproptosis-clusters was validated with the METABRIC data. The GO/KEGG analyses revealed that immune-related pathways were differentially enriched in the two cuproptosis-clusters, such as humoral immune response, cytokine–cytokine receptor interaction, and IL-17 signaling pathway. Further ESTIMATE and CIBERSORT analyses identified C1 as immune-excluded, with weakened immune cell infiltration, and C2 as immune-inflamed, with abundant infiltrating immune cells. The TIDE analysis indicated that patients in C2 could respond to immunotherapy better than patients in C1. A recent study identified copper as a factor that upregulates the expression of PD-L1 in tumor cells and modulates signaling pathways involved in PD-L1-mediated death (Voli et al., 2020), which might explain that C1 with higher expression levels of CRRs but be identified as immune-excluded to some extent.

We further developed and validated a CRPS for predicting the prognosis of BC. The low-risk group always had a better prognosis than the high-risk group. The ROC curves certified the reliability of the CRPS. The applicability of the CRPS was confirmed with the stratified analysis. We also found a close relationship between C2, the low-risk group, and the live event. To further explore the predictive ability of our signature, a comparison was performed among several significant molecular signatures employed for predicting OS in patients with BC. Compared with the other signature, such as ferroptosis, necroptosis, pyroptosis, and immune-related (Lin et al., 2020; Zhong et al., 2020; Wang et al., 2021a; Wu et al., 2021a; Zhu et al., 2021b; Ding et al., 2021; Ye et al., 2021; Lu et al., 2022a; Lu et al., 2022b; Chen et al., 2022; Chu et al., 2022; Yu et al., 2022; Zou et al., 2022), our signature indicated much higher AUCs, which indicated a better predictive ability, especially in predicting the long-term survival status. Furthermore, we constructed a prognostic nomogram that could simplify treatment decision-making for patients with BC.

Different tumor immune microenvironments characterized the two risk groups. The TIME was reported to play an essential role in BC, related to the therapeutic response and different clinical outcomes (Tower et al., 2019). The previous study indicated that TIME features were related to the OS in TNBC(Bareche et al., 2020). In the current study, the GSEA results implicated that immune-related pathways were enriched in the low-risk group. We further found that patients in the low-risk group had a higher immune score, estimate score and stromal score than the high-risk group. Furthermore, the low-risk group had an abundance infiltration of dendritic cells resting, activated NK cells, and CD8 T cells. Previous research has reported that these immune cells could protect against tumor growth and promote immune response and immunotherapy (Emens, 2012; Charoentong et al., 2017; Sato et al., 2018; Farhood et al., 2019). However, macrophages M2 were the main components in the high-risk group, which were the critical member in EMT and cancer metastasis (Biswas and Mantovani, 2010; Qian and Pollard, 2010). Furthermore, a series of typical ICPs, such as PD-L1, CTLA4 and HAVCR2, were significantly elevated in the low-risk group. Finally, according to the IPS and TIDE analyses, patients in the low-risk group tended to be the beneficiaries from immunotherapy. These findings suggested that the CRPS were associated with TIME and could guide targeted immunotherapies.

Although the CRPS showed the ability to predict prognosis potentially, there were several limitations to our study. Firstly, the regulatory mechanism of how cuproptosis affects the TIME in breast cancer was limited and deserved further research. In addition, *in vitro* and *in vivo* experiments were needed to confirm our findings.

## Conclusion

In conclusion, we firstly identified two cuproptosis-clusters in breast cancer with the different OS using consensus clustering. We further developed a cuproptosis-related prognostic signature that had good performance in predicting survival outcomes, tumor immune microenvironment and immunotherapy response for BC patients.

## Data Availability

Publicly available datasets were analyzed in this study. This data can be found here: https://xenabrowser.net/datapages/, http://www.cbioportal.org/, and http://www.ncbi.nlm.nih.gov/geo/.
