# Supplementary material for: The cuproptosis-related signature predicts prognosis and indicates immune microenvironment in breast cancer
Source: Front Genet. 2022 Sep 26;13:977322. doi: 10.3389/fgene.2022.977322 (PMC9548612; doi:10.3389/fgene.2022.977322)
Supplement: Supplementary file 1 [file DataSheet1.docx]

Supplementary Material


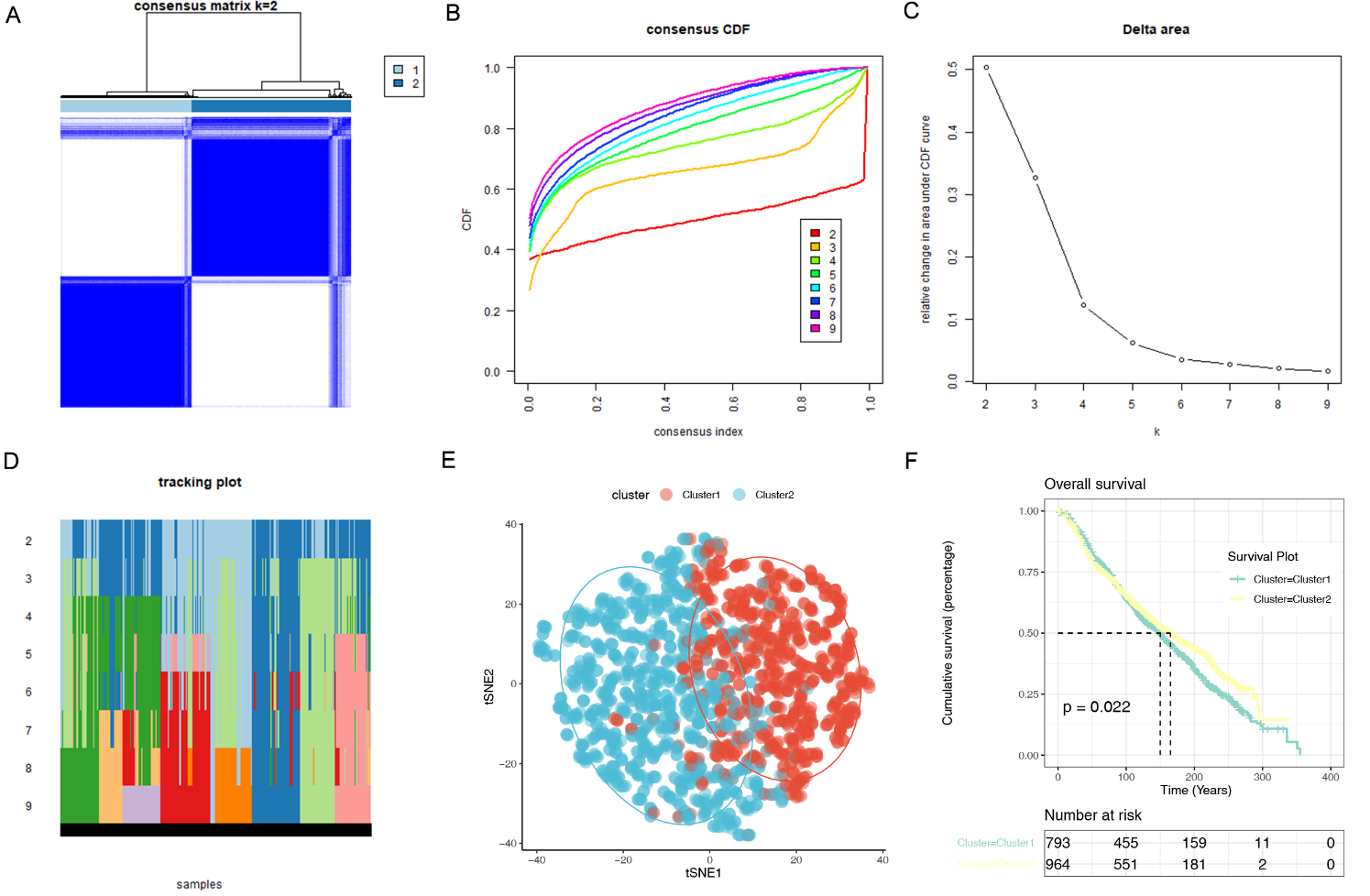


Supplementary Figure 1. Characterization of two cuproptosis-clusters based on CRRs in the METABRIC database. (A) Consensus matrix when k = 2. (B) Consensus CDF, (C) Delta area, (D) tracking plot, and tSNE plots (E) for validation of the clustering results. (F) Kaplan-Meier OS curves for BC patients between cluster 1 and cluster 2.


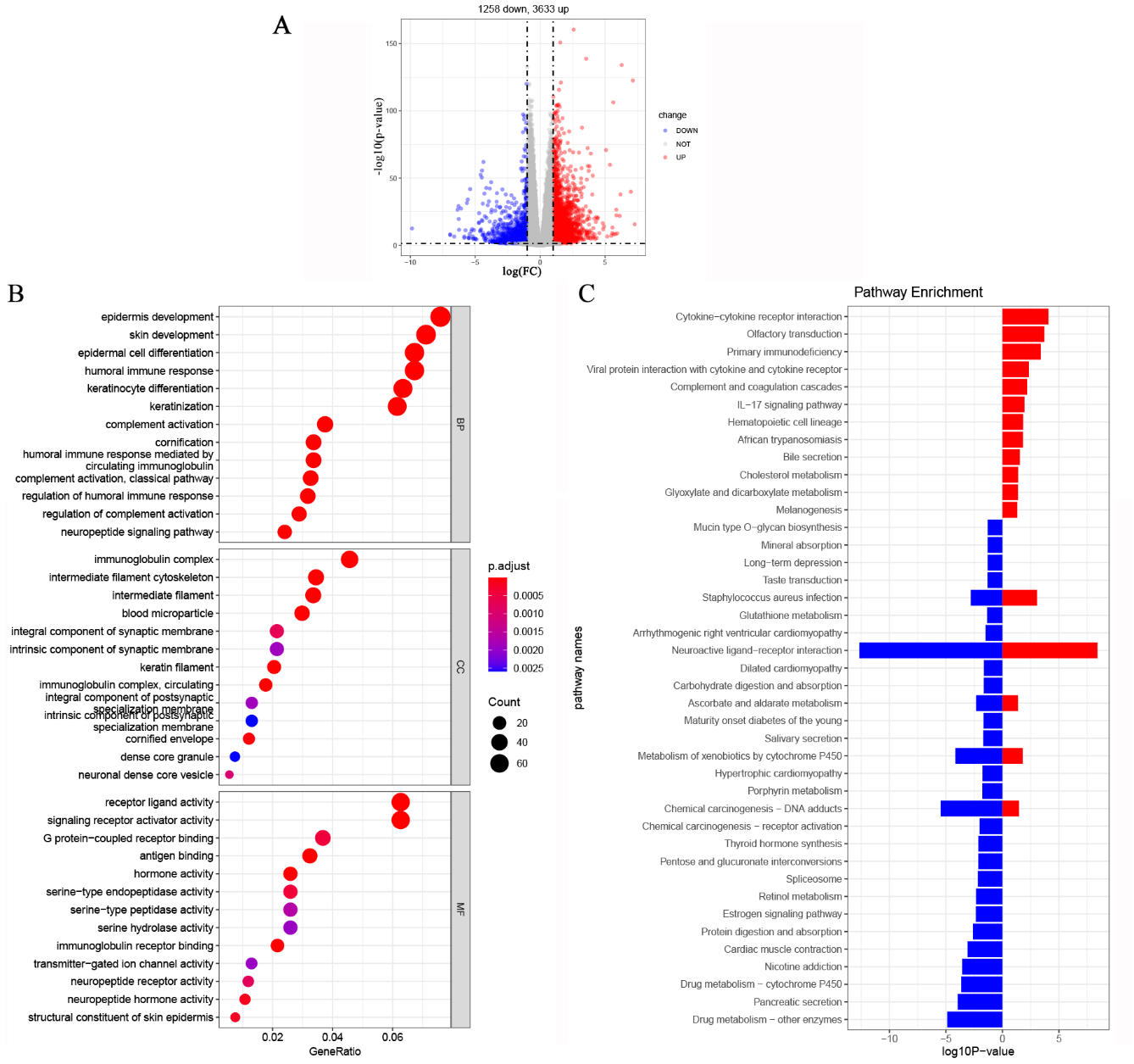


Supplementary Figure 2. The differential expression and function analyses between the two clusters. (A) The volcano plot of the DEGs between the two cuproptosis-clusters. GO (B) and KEGG (C) analyses of the DEGs.


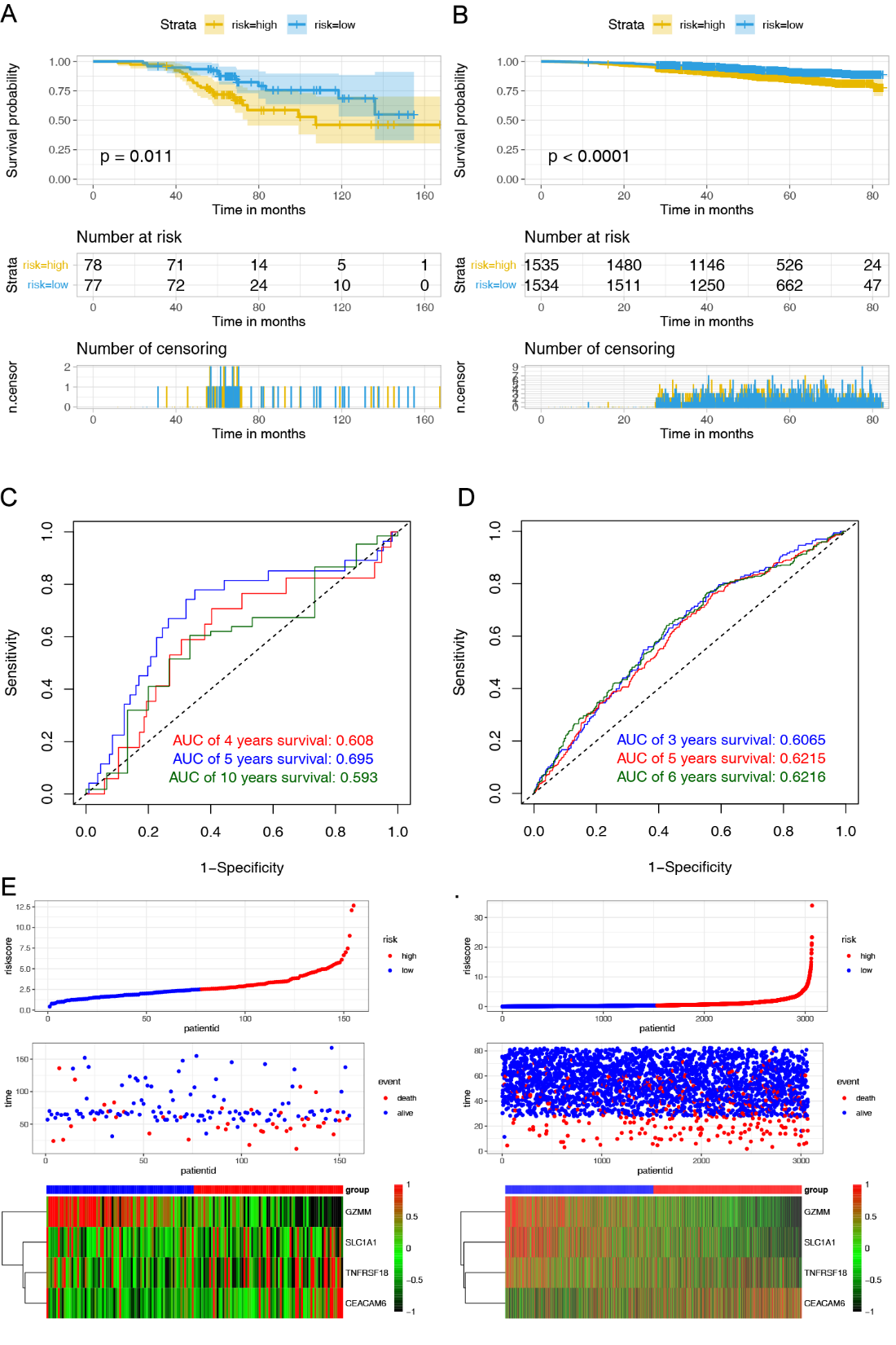


Supplementary Figure 3. Validation of the CRPS in the GSE9893 and GSE96058 databases. (A-B) Kaplan–Meier analyses of the OS between the two risk groups. (C-D) The 3-, 5- and 10-year ROC curves of the CRPS. (E-F) Ranked dot, scatter plots and the heat map of the model gene expressions.


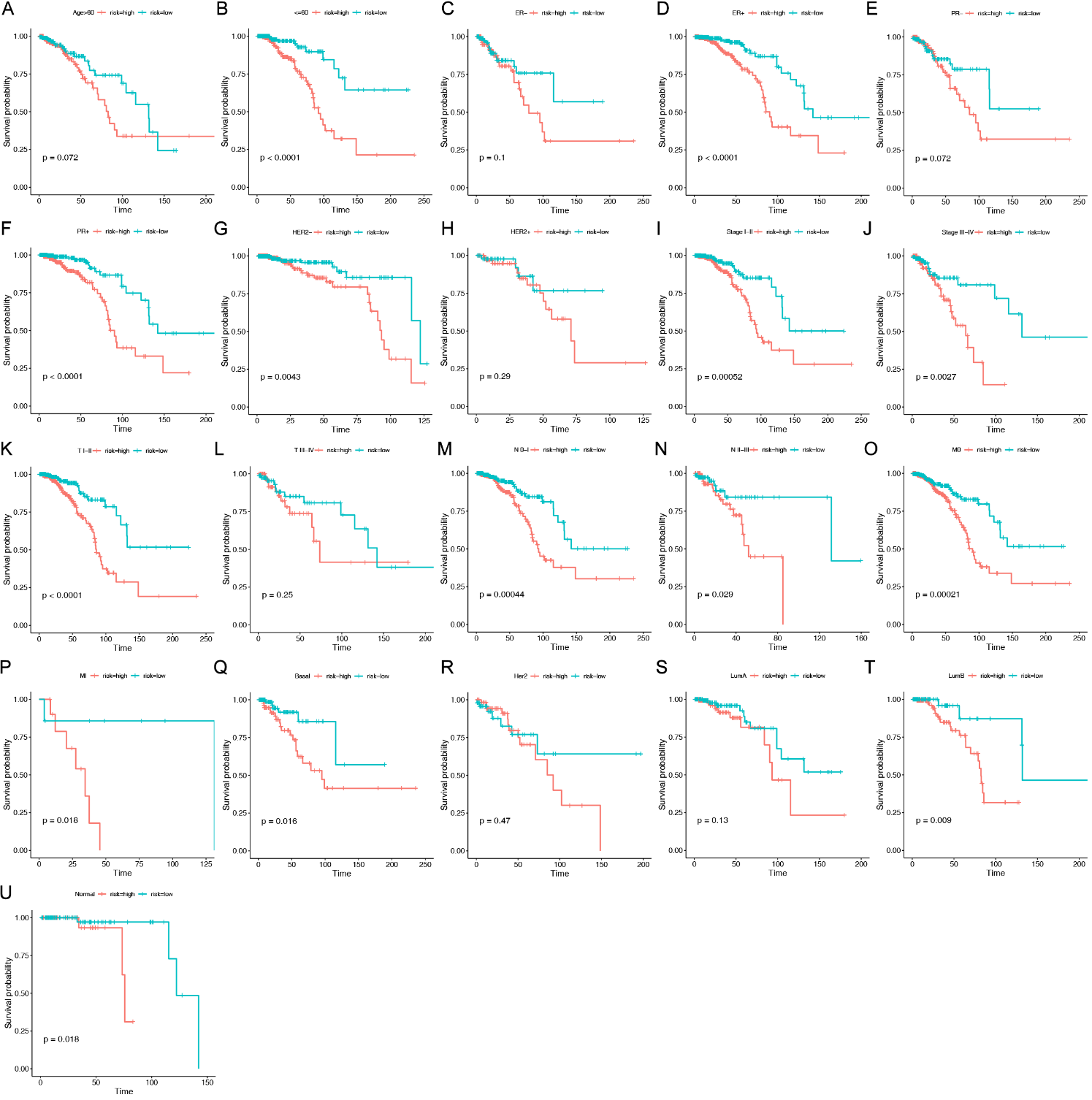


Supplementary Figure 4. Stratification analyses of the prognostic signature. Kaplan-Meier curves indicated the OS of the two risk groups stratified by age (>60 years vs. ≤60 years) (A, B), ER stage (negative vs. positive) (C, D), HER2 stage (negative vs. positive) (E, F), PR stage (negative vs. positive) (G, H), stages (stage I–II vs. stage III-IV) (I, J), AJCC T stage (T I–II vs. T III-IV) (K, L), AJCC N stage (N 0-I vs. T II-III) (M, N), AJCC M stage (M 0 vs. M I) (O, P), PAM50 subtypes (Basal, HER2, Luminal A, Luminal B and Normal-like), (Q-U), respectively.

| Characteristics | Type | Entire cohort (n=916) | Train cohort (n=458) | Test cohort (n=458) | Pvalue |
| --- | --- | --- | --- | --- | --- |
| age (%) | <=60 | 507 ( 55.3) | 259 ( 56.6) | 248 ( 54.1) | 0.765 |
|  | >60 | 409 ( 44.7) | 199 ( 43.4) | 210 ( 45.9) |  |
| Stage (%) | NA | 20 ( 2.2) | 14 ( 3.1) | 6 ( 1.3) | 0.736 |
|  | I | 169 ( 18.4) | 78 ( 17.0) | 91 ( 19.9) |  |
|  | II | 506 ( 55.2) | 256 ( 55.9) | 250 ( 54.6) |  |
|  | III | 203 ( 22.2) | 103 ( 22.5) | 100 ( 21.8) |  |
|  | IV | 18 ( 2.0) | 7 ( 1.5) | 11 ( 2.4) |  |
| T (%) | T1 | 254 ( 28.0) | 119 ( 26.3) | 135 ( 29.7) | 0.987 |
|  | T2 | 500 ( 55.1) | 254 ( 56.2) | 246 ( 54.1) |  |
|  | T3 | 121 ( 13.3) | 61 ( 13.5) | 60 ( 13.2) |  |
|  | T4 | 29 ( 3.2) | 16 ( 3.5) | 13 ( 2.9) |  |
|  | TX | 3 ( 0.3) | 2 ( 0.4) | 1 ( 0.2) |  |
| N (%) | N0 | 426 ( 47.0) | 203 ( 44.9) | 223 ( 49.0) | 0.561 |
|  | N1 | 308 ( 34.0) | 165 ( 36.5) | 143 ( 31.4) |  |
|  | N2 | 93 ( 10.3) | 42 ( 9.3) | 51 ( 11.2) |  |
|  | N3 | 65 ( 7.2) | 31 ( 6.9) | 34 ( 7.5) |  |
|  | NX | 15 ( 1.7) | 11 ( 2.4) | 4 ( 0.9) |  |
| M (%) | M0 | 746 ( 82.3) | 372 ( 82.3) | 374 ( 82.4) | 0.909 |
|  | M1 | 18 ( 2.0) | 7 ( 1.5) | 11 ( 2.4) |  |
|  | MX | 142 ( 15.7) | 73 ( 16.2) | 69 ( 15.2) |  |
| ER (%) | NA | 18 ( 2.0) | 11 ( 2.4) | 7 ( 1.5) | 0.745 |
|  | Indeterminate | 2 ( 0.2) | 2 ( 0.4) | 0 ( 0.0) |  |
|  | Negative | 200 ( 21.8) | 104 ( 22.7) | 96 ( 21.0) |  |
|  | Positive | 696 ( 76.0) | 341 ( 74.5) | 355 ( 77.5) |  |
| PR (%) | NA | 18 ( 2.0) | 11 ( 2.4) | 7 ( 1.5) | 0.942 |
|  | Indeterminate | 3 ( 0.3) | 1 ( 0.2) | 2 ( 0.4) |  |
|  | Negative | 289 ( 31.6) | 149 ( 32.5) | 140 ( 30.6) |  |
|  | Positive | 606 ( 66.2) | 297 ( 64.8) | 309 ( 67.5) |  |
| HER2 (%) | NA | 140 ( 15.3) | 71 ( 15.5) | 69 ( 15.1) | 0.755 |
|  | Equivocal | 159 ( 17.4) | 77 ( 16.8) | 82 ( 17.9) |  |
|  | Indeterminate | 12 ( 1.3) | 6 ( 1.3) | 6 ( 1.3) |  |
|  | Negative | 484 ( 52.8) | 254 ( 55.5) | 230 ( 50.2) |  |
|  | Positive | 121 ( 13.2) | 50 ( 10.9) | 71 ( 15.5) |  |

Supplementary Table 1. Clinical characteristic of the entire BC cohort, training cohort, and test cohort in TCGA database.

| id |
| --- |
| FDX1 |
| LIPT1 |
| LIAS |
| DLD |
| DBT |
| GCSH |
| DLST |
| DLAT |
| PDHA1 |
| PDHB |
| SLC31A1 |
| ATP7A |
| ATP7B |

Supplementary Table 2. The 13 cuproptosis-related regulators.

Supplementary Table 3. The univariate Cox regression analysis of the DEGs.

| characteristics | Hazard.Radio | CI95 | P.Value | CIL | CIU | HR.CI95 |
| --- | --- | --- | --- | --- | --- | --- |
| ZAP70 | 0.4861 | 0.2761-0.8558 | 0.012 | 0.2761 | 0.8558 | 0.4861 (0.2761-0.8558) |
| LTB | 0.6271 | 0.4437-0.8861 | 0.008 | 0.4437 | 0.8861 | 0.6271 (0.4437-0.8861) |
| KIAA1244 | 1.7463 | 1.0406-2.9304 | 0.035 | 1.0406 | 2.9304 | 1.7463 (1.0406-2.9304) |
| VPREB3 | 0.5906 | 0.3552-0.9819 | 0.042 | 0.3552 | 0.9819 | 0.5906 (0.3552-0.9819) |
| CYBA | 0.6158 | 0.4046-0.9374 | 0.024 | 0.4046 | 0.9374 | 0.6158 (0.4046-0.9374) |
| CLDN5 | 0.5999 | 0.3771-0.9541 | 0.031 | 0.3771 | 0.9541 | 0.5999 (0.3771-0.9541) |
| IGHG3 | 0.7558 | 0.6094-0.9373 | 0.011 | 0.6094 | 0.9373 | 0.7558 (0.6094-0.9373) |
| GZMM | 0.488 | 0.2944-0.809 | 0.005 | 0.2944 | 0.809 | 0.488 (0.2944-0.809) |
| CEACAM6 | 1.2252 | 1.0256-1.4637 | 0.025 | 1.0256 | 1.4637 | 1.2252 (1.0256-1.4637) |
| SLC1A1 | 0.7041 | 0.5207-0.9521 | 0.023 | 0.5207 | 0.9521 | 0.7041 (0.5207-0.9521) |
| MATK | 0.4017 | 0.2069-0.7801 | 0.007 | 0.2069 | 0.7801 | 0.4017 (0.2069-0.7801) |
| APOE | 0.647 | 0.4591-0.9117 | 0.013 | 0.4591 | 0.9117 | 0.647 (0.4591-0.9117) |
| PTGDS | 0.6952 | 0.4869-0.9925 | 0.045 | 0.4869 | 0.9925 | 0.6952 (0.4869-0.9925) |
| IGKC | 0.7737 | 0.6419-0.9324 | 0.007 | 0.6419 | 0.9324 | 0.7737 (0.6419-0.9324) |
| TNFRSF18 | 0.635 | 0.4192-0.9619 | 0.032 | 0.4192 | 0.9619 | 0.635 (0.4192-0.9619) |
| MRPL12 | 0.5799 | 0.3567-0.9428 | 0.028 | 0.3567 | 0.9428 | 0.5799 (0.3567-0.9428) |
